# A Post-GWAS Functional Analysis Confirming Effects of Three BTA13 Genes *CACNB2*, *SLC39A12*, and *ZEB1* on Dairy Cattle Reproduction

**DOI:** 10.3389/fgene.2022.882951

**Published:** 2022-06-08

**Authors:** Abdul Sammad, Hailiang Zhang, Rui Shi, Yixin Dong, Hanpeng Luo, Ziwei Chen, Lin Liu, Gang Guo, Aoxing Liu, Yachun Wang

**Affiliations:** ^1^ Key Laboratory of Animal Genetics, Breeding and Reproduction, MARA, National Engineering Laboratory for Animal Breeding, College of Animal Science and Technology, China Agricultural University, Beijing, China; ^2^ Beijing Dairy Cattle Center, Beijing, China; ^3^ Beijing Sunlon Livestock Development Co. Ltd., Beijing, China; ^4^ Center for Quantitative Genetics and Genomics, Aarhus University, Aarhus, Denmark

**Keywords:** Holstein, fertility, gene expression, ovarian follicle, haplotype

## Abstract

In our previous GWAS of Chinese and Nordic dairy cattle, genes *CACNB2*, *SLC39A12,* and *ZEB1* locating on BTA 13 were suggested as candidate genes for reproduction. In this study, validation of these associations was performed in an independent population with records of nine reproductive traits. More importantly, functions of these genes in the reproductive process were verified by employing the expression data of ovarian follicles. The potential variants within the three genes were firstly detected in 68 Chinese Holstein bulls, and then screened in 1,588 Chinese Holstein cows using the KASP (Kompetitive allele-specific PCR) method. There were nine variants with polymorphisms in *CACNB2*, five in *SLC39A12,* and four in *ZEB1*, respectively, of which one SNP was in the upstream regulatory region, two in exon region, four in downstream regulatory region, and 11 SNPs in intronic regions. Amongst the 18 variants, g.33267056T/G in *CACNB2* explained the largest phenotypic variance for age at first calving (0.011%), interval from first to last insemination (0.004%), and calving ease (0.002%), while g.32751518G/A in *SLC39A12* contributed the most to stillbirth in heifers (0.038%). Two haplotype blocks were constructed for *CACNB2* while one each for *SLC39A12* and *ZEB1*, which were significantly associated with five reproductive traits, including age at the first service, age at the first calving, calving ease in heifers and cows, and the interval from calving to the first insemination. We then studied the profile of gene expression in granulosa cells isolated from four developmental stages of ovarian follicles from eight dairy cows. All three genes were differentially expressed between ovarian follicles with different sizes (*p* < 0.05), indicating their potential roles in the reproductive process of dairy cows. This study successfully demonstrated the associations of three BTA 13 genes *CACNB2*, *SLC39A12,* and *ZEB1* with reproduction and further examined their expression levels in ovarian follicles directly. These findings can be beneficial for the ongoing genomic selection program for reproductive traits which have long been considered as traits that are difficult to achieve genetic improvement due to the lack of efficient genetic markers.

## 1 Introduction

Poor reproductive performances of dairy heifers and cows can largely reduce the efficiency and overall profitability of dairy herds due to extra costs on herd management, veterinary care, and involuntary culling (Dobson et al., 2007; Guo et al., 2014; Liu et al., 2017a). This makes reproduction among the most important functional traits in dairy industry globally. However, due to the highly polygenic character and its negative genetic correlations with intensively selected milk production traits, genetic improvement of reproductive performance is relatively slow (Dobson et al., 2007; Berry et al., 2014). By integrating causal mutations or variants that are in tight LD (linkage disequilibrium) with causal mutations into genomic prediction, the prediction reliability of breeding values can be improved (Schultz et al., 2020), and therefore, accelerating the dairy cattle genetic improvement program.

Genome-wide association study (GWAS) has been served as the primary strategy to detect genes associated with complex traits in the past 15 years. In our previous GWAS of Chinese and Nordic Holsteins (Liu et al., 2017b), we were able to detect QTLs (quantitative trait loci) for reproductive traits on BTA 13 and concluded genes *CACNB2* (calcium voltage-gated channel auxiliary subunit beta 2, for interval from calving to the first insemination (ICF) and interval from first to last insemination in cows (IFLc)), *SLC39A12* (solute carrier family 39 member 12, for IFLc), and *ZEB1* (zinc finger E-box binding homeobox 1, for ICF and IFLc) as candidate genes. The gene *CACNB2* encodes the β subunit of voltage-gated calcium channels (Ca+), which is a ubiquitous secondary messenger that regulates most cellular processes and also a prerequisite for gonadotropin-releasing hormone receptors (GnRH)-induced activation of extracellular signal-regulated kinase that medicates the secretion of FSH (Clapham, 2007; Parekh, 2011). Another GWAS in Japanese Holsteins also identified *CACNB2* as a candidate gene for conception rate and addressed its role in releasing follicle-stimulating hormone from the anterior pituitary gland (Sugimoto et al., 2013). However, the causative SNPs within *CACNB2* for reproduction is still not fine confirmed; moreover, in addition to conception rate, its effects on other reproductive traits remain to be investigated. The gene *SLC39A12* is a protein-coding gene and involves in the transport of zinc into the cytoplasm. The protein coded by *SLC39A12* belongs to the subgroup of proteins that shows the structural characteristics of zinc transporters and serves as an important component of the internal homeostasis of the cellular zinc (Taylor and Nicholson, 2003; Lisle et al., 2013). The gene *ZEB1*, encoding zinc finger and homologous domain transcription factors, is one of the key transition factors of epithelial mesenchymal transition in epithelial membrane cells (Bilyk et al., 2017). However, there is a lack of functional analyses to confirm the effect of gene *SLC39A12* and *ZEB1* on reproduction in cattle.

Our previous GWAS in Chinese and Nordic Holsteins used the 54K SNP array that was designed to cover common SNPs in main cattle breeds to achieve acceptable performances of genomic prediction (Matukumalli et al., 2009). The variants with the most significant effect reported in GWAS are therefore not necessary to be causal for having a direct impact on reproduction, given the limitation in genotyping resolution. Although the use of multiple Holstein populations can narrow down QTL intervals to some extent, the QTL reported in GWAS was still extensively large because LD structures are generally consisted within the Holstein breed (Zhou et al., 2013). In addition, there could be multiple independent loci associated with reproductive traits within a gene. Although some significant associations (e.g., *CACNB2*) were able to be replicated in populations other than Chinese and Nordic Holsteins, the post-GWAS analyses are still necessary to narrow down QTLs and even fine-map causative mutations.

Post-GWAS screening and whole gene sequencing are capable to increase the resolution of variant associations discovered by GWAS (Visscher et al., 2017). One widely used strategy for post-GWAS is to highlight the role of coding variants (e.g., missense variants) via exome sequencing (Zhao et al., 2015). Additionally, genetic variations in non-coding regions (e.g., intron) can be missed by GWAS when using commercial SNP arrays although they could still explain part of the phenotypic variations due to the functional relevance to causal variants (Cooper, 2010; Lomelin et al., 2010). The primary objective of post-GWAS analysis is to better understand the physiological pathway of the target trait represented by the candidate genes at the molecular level (Emig and Albrecht, 2011). The mRNA expression analyses have already been used to validate GWAS findings for several complex traits in dairy cattle such as milk production traits (Han et al., 2019), mastitis (Mi et al., 2021), and heat stress resistance (Fang et al., 2021). However, post-GWAS studies on reproduction traits in dairy cattle is relatively scarce. One main reason could be the difficulty to collect relevant tissue samples (Struck et al., 2018). For studying reproductive performances, ovarian follicles can be perfect samples due to its indispensable roles in reproduction process. Furthermore, a strong genotyping cohort and powerful population data used in the current study is suggestive of reliable results (Visscher et al., 2017).

Collectively, the objectives of this study were to explore the effects of three BTA 13 candidate genes *CACNB2*, *SLC39A12,* and *ZEB1* on dairy cattle reproduction, by employing haplotype analyses and gene expression data. The findings from this study could positively contribute to the genetic improvement of reproduction performances in dairy cattle.

## 2 Methods

**FIGURE 1:**
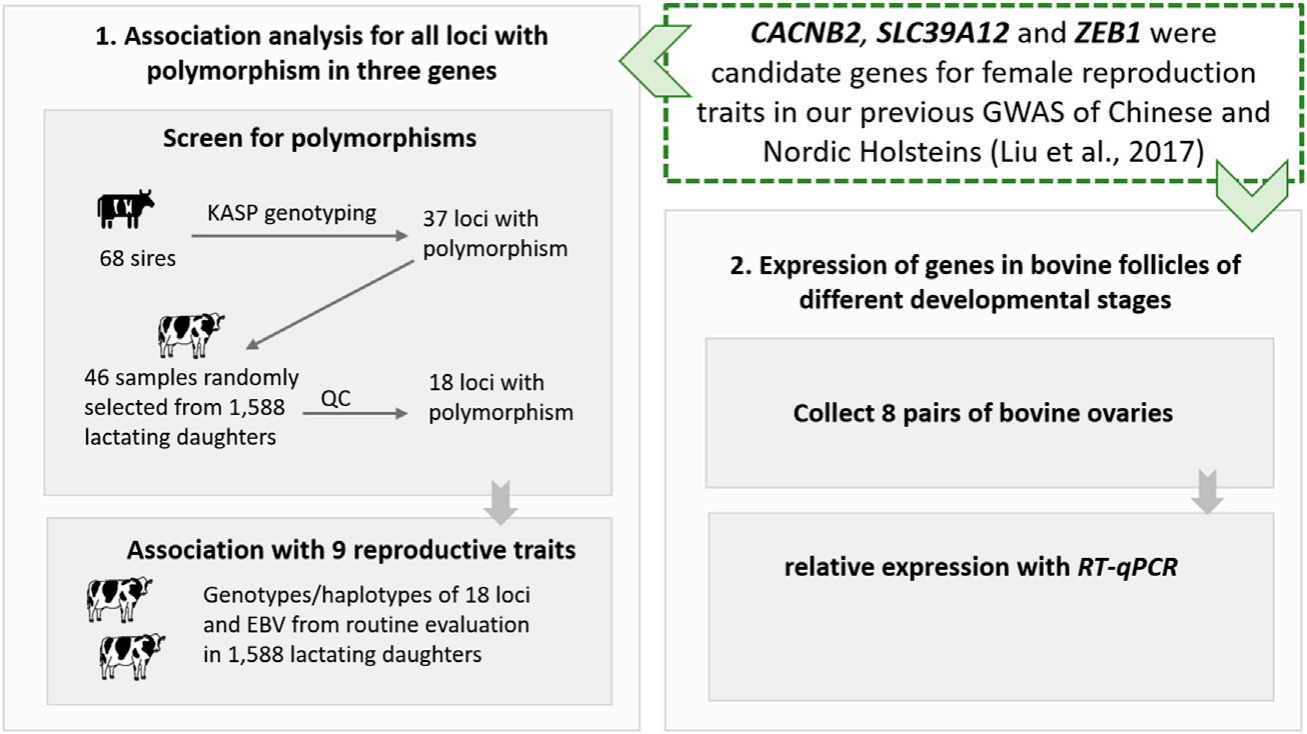
The designed technology route of post-GWAS functional analysis for gene *CACNB2*, *SLC39A12* and *ZEB1* in Holstein cattle.

In this study, to explore the roles of three GWAS candidate genes (*CACNB2*, *SLC39A12,* and *ZEB1*) on dairy cattle reproduction, we first screened for polymorphisms and performed genotype- and haplotype-based association analyses in an independent Holstein population, and then studied the profile of gene expression in granulosa cells isolated from 8 cows. The designed technology route of this study is shown in Figure 1.

### 2.1 Genotype and Haplotype-Based Association Analysis

#### 2.1.1 Screen for Polymorphisms in 68 Bulls

Premier 5.0 and Oligo 7.0 (Sangon Biotech Co., Ltd. Shanghai) were used to design primes for all exons and flanking regions (within 2 kb distance of 5′ and 3’ end) in three GWAS candidate genes. In total, 50 pairs of primers (Supplementary Table S1) were designed, including 16 for *CACNB2*, 18 for *SLC39A12,* and 16 for *ZEB1*. These primers were then synthesized at Sangon Biotech (Shanghai, China).

The DNA samples were extracted from frozen semen of 68 Holstein bulls (Beijing Dairy Cattle Center, Beijing, China) using a standard phenol-chloroform method, and then, DNA concentration were diluted to 200 ng/μl by adding TB buffer. We randomly mixed DNA samples into three pools (one pool contained samples from 28 bulls and two pools contained 20 samples each) and then used pooled samples for the subsequent PCR amplification [ABI3730XL DNA analyzer (Applied Biosystems, Foster, CA, United States)]. Sequencing data were aligned to the reference genome (UMD 3.1) using Ensemble (https://asia.ensembl.org/) and examined for potential polymorphism using Chromas (http://technelysium.com.au/wp/chromas/) and DNAman (https://www.lynnon.com/dnaman.html/). In total, 37 variants were detected from genes *CACNB2*, *SLC39A12,* and *ZEB1*.

#### 2.1.2 Genotype and Phenotype of 1,588 Cows

We considered 1,588 Holstein cows from eight dairy farms (Beijing Sunlon Livestock Development Co. Ltd, Beijing, China), of which complete pedigree and reproductive information have been recorded. All cows were elaborately selected to be independent of the GWAS discovery population (Liu et al., 2017b). DNA samples of cows were extracted from the whole blood samples using TIANamp Blood DNA Kit (TIANgen, Beijing, China). To shortlist variants included for KASP (Kompetitive allele-specific PCR) genotyping, a pilot analysis was performed in 46 (out of 1,588) randomly selected samples. Only one variant was kept if the distance between two variants was less than 200 bp. Ultimately, 18 variants (Table 1) were remained and used to genotype all 1,588 cows with the KASP method. A chi-square test was used to determine whether allelic frequencies of any variant were deviated from Hardy-Weinberg equilibrium. Bonferroni correction was applied to control for familywise false positive.

**TABLE 1:** The detailed information of 18 SNPs within the gene *CACNB2*, *SLC39A12* and *ZEB1* in 1,588 Holstein cows.

Gene	SNP	Genotype	Number of individuals	Genotypic Frequency	Allele	Allelic Frequency	H-W-E Test, χ2 Value (df = 1)
*CACNB2*	g.33253706A/C	A:A	462	0.312	A	0.559	
		C:A	735	0.496	C	0.441	0.835
		C:C	286	0.193			
*CACNB2*	g.33284577T/C	C:C	266	0.181	C	0.431	
		C:T	739	0.502	T	0.569	0.385
		T:T	468	0.318			
*CACNB2*	g.33258042G/T	G:G	430	0.294	G	0.546	
		T:G	739	0.505	T	0.454	0.466
		T:T	294	0.201			
*CACNB2*	g.33258138T/G	G:G	315	0.216	G	0.471	
		G:T	743	0.510	T	0.529	0.386
		T:T	400	0.274			
*CACNB2*	g.33258186G/A	A:A	146	0.102	A	0.322	
		A:G	628	0.440	G	0.678	0.782
		G:G	653	0.458			
*CACNB2*	g.33258354A/G	A:A	407	0.278	A	0.530	
		G:A	738	0.504	G	0.470	0.629
		G:G	318	0.217			
*CACNB2*	g.33267056T/G	G:G	223	0.188	G	0.436	
		G:T	590	0.496	T	0.564	0.753
		T:T	376	0.316			
*CACNB2*	g.33267172C/T	C:C	657	0.460	C	0.677	
		T:C	619	0.433	T	0.323	0.730
		T:T	152	0.106			
*CACNB2*	g.33267296G/C	C:C	64	0.044	C	0.213	
		C:G	490	0.338	G	0.787	0.772
		G:G	896	0.618			
*SLC39A12*	g.32664855G/A	A:A	55	0.038	A	0.191	
		A:G	448	0.307	G	0.809	0.776
		G:G	957	0.655			
*SLC39A12*	g.32665313G/A	A:A	929	0.642	A	0.801	
		A:G	461	0.318	G	0.199	0.932
		G:G	58	0.040			
*SLC39A12*	g.32698687A/G	A:A	2	0.001	A	0.056	
		G:A	163	0.110	G	0.944	0.185
		G:G	1,313	0.888			
*SLC39A12*	g.32751518G/A	A:A	225	0.154	A	0.393	
		A:G	700	0.479	G	0.607	0.900
		G:G	537	0.367			
*SLC39A12*	g.32668290G/A	A:A	13	0.009	A	0.106	
		A:G	288	0.195	G	0.894	0.311
		G:G	1,175	0.796			
*ZEB1*	g.34110507T/A	A:A	481	0.418	A	0.647	
		T:A	529	0.459	T	0.353	0.853
		T:T	142	0.123			
*ZEB1*	g.34061171T/C	A:A	40	0.028	A	0.135	
		G:A	302	0.214	G	0.865	0.001
		G:G	1,069	0.758			
*ZEB1*	g.34066997C/G	C:C	277	0.190	C	0.430	
		C:G	697	0.479	G	0.570	0.386
		G:G	481	0.331			
*ZEB1*	g.34063562C/G	C:C	292	0.201	C	0.440	
		C:G	692	0.477	G	0.560	0.229
		G:G	466	0.321			

We explored the relationship of 18 variants with nine reproductive traits which covered the broad mechanisms of cattle reproduction (Ettema and Santos, 2004; Nayeri et al., 2017), including age at the first service (AFS) and calving (AFC), the interval from calving to the first insemination (ICF), the interval from the first to last insemination in heifers (IFL_H) and cows (IFL_C), stillbirth in heifers (SB_H) and cows (SB_C), and calving ease in heifers (CE_H) and cows (CE_C). Estimated breeding values (EBVs), derived from single-trait animal models (Liu et al., 2017a) by national routine genetic evaluation (Independent Innovation League of Dairy Breeding, Beijing, China), were used as the response variable for association analyses. Descriptive statistics of EBVs for each reproductive trait were presented in Supplementary Table S2.

#### 2.1.3 Construct Haplotypes Based on LD Structures

For each candidate gene, haplotype blocks were constructed based on LD structures of identified SNPs by using the Haploview4.0 software (Gabriel et al., 2002; Barrett et al., 2005). Haplotypes with relative frequency less than 5% were merged into one group (Table 2).

**TABLE 2:** Association of haplotype blocks within gene *CACNB2*, *SLC39A12* and *ZEB1* with reproductive traits in Holstein cows (Least Square Mean ± Standard Error).

Haplotype	AFC^1^	AFS	CE_C	CE_H	ICF	IFL_C	IFL_H	SB_C	SB_H
*CACNB2*-Block 1									
H1H1(428)	−10.092 ± 0.309b	−17.819 ± 0.374	0.002 ± 0.000a	0.008 ± 0.001	0.500 ± 0.054	2.019 ± 0.247	6.388 ± 0.303	0.014 ± 0.001	0.029 ± 0.002
H1H2(702)	−8.882 ± 0.241a	−17.333 ± 0.292	0.000 ± 0.000b	0.008 ± 0.000	0.366 ± 0.042	2.596 ± 0.192	6.737 ± 0.237	0.015 ± 0.000	0.029 ± 0.001
H1H3(20)	−7.849 ± 1.429ab	−15.787 ± 1.730	0.001 ± 0.001ab	0.008 ± 0.003	0.596 ± 0.251	3.499 ± 1.140	8.042 ± 1.402	0.017 ± 0.003	0.025 ± 0.007
H2H2(266)	−8.856 ± 0.392ab	−17.143 ± 0.474	0.000 ± 0.000b	0.010 ± 0.001	0.272 ± 0.069	2.825 ± 0.313	6.838 ± 0.384	0.015 ± 0.001	0.024 ± 0.002
H2H3(25)	−8.745 ± 1.278ab	−19.132 ± 1.548	0.000 ± 0.001ab	0.006 ± 0.002	0.452 ± 0.224	4.359 ± 1.020	7.747 ± 1.254	0.018 ± 0.003	0.040 ± 0.006
*p* value	0.019	0.455	0.001	0.121	0.088	0.057	0.589	0.316	0.077
*CACNB2*-Block 2									
H1H1(306)	−10.287 ± 0.365b	−18.365 ± 0.456	0.002 ± 0.000a	0.009 ± 0.001ab	0.505 ± 0.063	2.068 ± 0.290	6.375 ± 0.359	0.015 ± 0.001	0.028 ± 0.002
H1H2(390)	−8.570 ± 0.323a	−16.695 ± 0.404	0.000 ± 0.000ab	0.008 ± 0.001ab	0.438 ± 0.056	2.601 ± 0.257	7.460 ± 0.318	0.015 ± 0.001	0.028 ± 0.002
H1H3(121)	−9.802 ± 0.580ab	−18.569 ± 0.725	0.000 ± 0.000abc	0.005 ± 0.001b	0.303 ± 0.101	3.298 ± 0.461	5.986 ± 0.571	0.016 ± 0.001	0.027 ± 0.003
H1H4(51)	−9.471 ± 0.894ab	−17.435 ± 1.117	0.003 ± 0.001a	0.009 ± 0.002ab	0.581 ± 0.155	1.358 ± 0.710	5.370 ± 0.880	0.017 ± 0.002	0.028 ± 0.004
H2H2(118)	−8.917 ± 0.588ab	−18.013 ± 0.734	0.000 ± 0.000bc	0.011 ± 0.001a	0.421 ± 0.102	3.209 ± 0.467	7.666 ± 0.579	0.016 ± 0.001	0.035 ± 0.003
H2H3(71)	−7.076 ± 0.757a	−17.160 ± 0.947	-0.002 ± 0.001c	0.006 ± 0.001ab	0.187 ± 0.131	3.328 ± 0.602	7.596 ± 0.746	0.015 ± 0.002	0.020 ± 0.004
H2H4(24)	−7.101 ± 1.303ab	−14.107 ± 1.628	0.001 ± 0.001abc	0.010 ± 0.003ab	0.479 ± 0.226	2.946 ± 1.035	7.240 ± 1.283	0.015 ± 0.003	0.016 ± 0.006
H3H3(14)	−7.594 ± 1.706ab	−12.628 ± 2.132	0.001 ± 0.001abc	0.008 ± 0.003ab	0.217 ± 0.296	3.587 ± 1.355	7.817 ± 1.680	0.016 ± 0.003	0.022 ± 0.008
*p* value	0.002	0.011	<0.0001	0.042	0.431	0.086	0.083	0.962	0.054
*SLC39A12*-Block									
H1H1(686)	−9.487 ± 0.244	−17.531 ± 0.294	0.001 ± 0.000ab	0.008 ± 0.000	0.448 ± 0.043	2.411 ± 0.196	6.801 ± 0.239	0.015 ± 0.000	0.029 ± 0.001
H1H2(379)	−8.624 ± 0.328	−17.830 ± 0.395	0.000 ± 0.000b	0.008 ± 0.001	0.294 ± 0.058	2.752 ± 0.263	6.598 ± 0.322	0.015 ± 0.001	0.029 ± 0.002
H1H3(215)	−8.978 ± 0.436	−16.615 ± 0.525	0.001 ± 0.000ab	0.010 ± 0.001	0.436 ± 0.077	2.803 ± 0.349	6.608 ± 0.428	0.015 ± 0.001	0.029 ± 0.002
H2H2(55)	−9.581 ± 0.862	−16.965 ± 1.038	0.000 ± 0.001ab	0.010 ± 0.002	0.241 ± 0.152	2.454 ± 0.691	6.201 ± 0.845	0.013 ± 0.002	0.024 ± 0.004
H2H3(58)	−9.047 ± 0.839	−18.654 ± 1.010	0.000 ± 0.001ab	0.007 ± 0.002	0.327 ± 0.148	2.178 ± 0.673	6.428 ± 0.823	0.013 ± 0.002	0.023 ± 0.004
H3H3(12)	−9.634 ± 1.846	−15.943 ± 2.221	0.002 ± 0.002a	0.011 ± 0.004	0.143 ± 0.325	1.495 ± 1.479	5.736 ± 1.810	0.017 ± 0.004	0.030 ± 0.009
*p* value	0.422	0.347	0.032	0.174	0.246	0.783	0.960	0.790	0.616
*ZEB1*-Block									
H1H1(455)	−9.592 ± 0.303	−17.439 ± 0.366	0.001 ± 0.000	0.008 ± 0.001	0.468 ± 0.053	2.521 ± 0.241	6.755 ± 0.296	0.014 ± 0.001	0.031 ± 0.001
H1H2(457)	−9.113 ± 0.302	−17.359 ± 0.365	0.001 ± 0.000	0.009 ± 0.001	0.501 ± 0.053	2.405 ± 0.241	7.022 ± 0.295	0.014 ± 0.001	0.028 ± 0.001
H1H3(178)	−9.406 ± 0.484	−18.160 ± 0.585	0.000 ± 0.000	0.007 ± 0.001	0.246 ± 0.084	2.923 ± 0.385	5.943 ± 0.473	0.016 ± 0.001	0.027 ± 0.002
H2H2(110)	−9.098 ± 0.616	−16.798 ± 0.744	0.000 ± 0.001	0.009 ± 0.001	0.253 ± 0.107	1.988 ± 0.490	6.998 ± 0.602	0.016 ± 0.001	0.029 ± 0.003
H2H3(111)	−8.299 ± 0.613	−18.082 ± 0.741	0.000 ± 0.001	0.007 ± 0.001	0.166 ± 0.107	2.987 ± 0.488	6.175 ± 0.599	0.016 ± 0.001	0.024 ± 0.003
H3H3(38)	−7.275 ± 1.048	−15.450 ± 1.266	0.000 ± 0.001	0.013 ± 0.002	0.092 ± 0.182	3.169 ± 0.834	7.196 ± 1.024	0.015 ± 0.002	0.029 ± 0.005
*p* value	0.187	0.355	0.018	0.111	0.003	0.552	0.414	0.159	0.435

1 AFC, age at the first service; AFS, age at the first calving; CE_C, calving ease in cows; CE_H, calving ease in heifers; ICF, the interval from calving to the first insemination; IFL_C, the interval from the first to last insemination in cows; IFL_H, the interval from the first to last insemination in heifers; SB_C, stillbirth in cows; SB_H, stillbirth in heifers.

#### 2.1.4 Association Analysis

Association analyses between every identified SNP or haplotype block and each reproductive trait were performed by using the GLM procedure of SAS 9.2. For both SNP- and haplotype block-based analyses, Bonferroni correction was used to control for false positives resulting from multiple testing. The model used for association analysis of each reproductive trait is as follows:

y=µ+G+e

Where y is individual’s EBV; µ is the overall mean; G is the fixed effect of either genotype or haplotype block; and e is the random residual effect. The proportion of the phenotypic variance $\left(\sigma_{P}^{2}\right)$ explained by each identified SNP was calculated as $\left(\frac{\sigma_{g}^{2}}{\sigma_{P}^{2}}\right)$ , where $\sigma_{g}^{2}$ is the variance explained by SNP (Hill and Mackay, 2004) and $\sigma_{P}^{2}$ is the phenotypic variance (Supplementary Table S2).

### 2.2 Gene Expression Assay of *CACNB2*, *SLC39A12,* and *ZEB1*

#### 2.2.1 Ovary Collection, Follicle Selection, and Granulosa Cell Isolation

Bovine ovaries of eight dairy cows were collected at an abattoir and put into thermally insulated bottles (28–30°C) containing sterile physiological saline (with 100 U/ml Penicillin and 0.1 mg/ml Streptomycin) immediately. In less than 2 hours, ovaries were transported to the laboratory. After washing three times with warm (37.5°C) 0.9% NaCl solution and rinsing in 70% warm ethanol for 30 s, ovaries were washed with Dulbecco’s Phosphate-Buffered Saline (DPBS) for three times. Healthy ovarian follicles with amber-colored fluid were kept and classified into four developmental stages according to their diameters (d), including stage 1 (d ≤ 3 mm), 2 (3 mm < d ≤ 7 mm), 3 (7 mm < d ≤ 10 mm), and 4 (d > 10 mm), as shown by Figure 4A. The follicular fluid contained both cumulus-oocyte complexes (COCs) and granulosa cells (GCs). We used a filter (diameter = 70 μm) to filter out COCs and then centrifuged the filtrates (contained GCs) at 1,500 × *g* for 5 min. The supernatant of the follicular fluid was discarded by aspiration. The GC cells were washed three times in phosphate-buffered saline (pH 7.4) and placed in DMEM/F-12 (Gibco, Life Technologies Inc., Grand Island, NY, United States) which contained 1% penicillin-streptomycin and 10% fetal bovine serum (FBS, Gibco, Life Technologies Inc., Grand Island, NY, United States).

#### 2.2.2 Quantitative Reverse Transcription PCR

The RT-qPCR was conducted to capture the relative expression level of genes *CACNB2*, *SLC39A12,* and *ZEB1* in GCs collected from ovarian follicles at different developmental stages. Total RNA was extracted from three biological replicates of GCs from follicles with different stages. Reverse transcription was carried out through first-strand cDNA synthesis kit (Thermo Fisher Scientific, Germany) with oligo (dT) 18 primers according to the manufacturer protocols. Primer 3 (version 4.0, http://bioinfo.ut.ee/primer3/) and Primer blast (http://www.ncbi.nlm.nih.gov/tools/primer-blast/) was used to design gene-specific primers spanning the exons (Supplementary Table S3).

The RT-qPCR was carried out through iTaq™ universal SYBR^®^ Green Super-mix (Bio-Rad Laboratories GmbH, Germany) in Applied Biosystem^®^ Step-OnePlus™ (Ap-plied Biosystems, CA, United States). A reaction volume of 20 µl was used, including 7.4 µl of double-distilled H2O, 0.3 µl of forwarding primer, 0.3 µl of reverse primer, 10 µl of 1x SYBR Green master mix (Bio-Rad Laboratories GmbH, Germany), and 2 µl of cDNA template. Light Cycler 480 instrument (Roche, Germany) was used to perform qPCR. The second derivative maximum method was employed for data acquiring. Although its reliability as a housekeeping gene in GCs has not been validated in this study, the gene *GAPDH* was used as the reference gene in RT-qPCR. The CT value adjusted by reference gene was used to measure the gene expression level at each developmental stage. The ANOVA procedure applied in the SAS software was used to test the differences in relative mRNA expression levels among four developmental stages. Bonferroni *t* test was used to perform multiple comparisons. The relative mRNA expression levels of each gene at different developmental stages were presented with least square means.

## 3 Results

### 3.1 Polymorphism Screening

In this study, by using 68 bulls, a total of 37 variants were identified from polymorphism screening, including 13 in *CACNB2*, 10 in *SLC39A12*, and 14 in *ZEB1*. Although most genetic variants of dairy cattle are from the gene pool of bull, some variants presenting in bulls may be absent or with poor polymorphism in cow population. To efficiently perform KASP genotyping in 1,588 cows for target SNPs with variants, we performed a pilot analysis conducting of 46 cows. Some loci without polymorphism or with poor polymorphism in cow population was firstly filtered out, and the variants that do not meet primer design criteria of KASP are also removed. Furthermore, considering the high LD, only one SNP within 200 bp was remained for association analysis. From the pilot analysis using 46 selected samples, 18 variants with polymorphism in gene *CACNB2* (nine), *SLC39A12* (five) and *ZEB1* (four) were further genotyped in 1,558 Holstein cows. The allele frequencies and the corresponding *p*-values derived from the Hardy-Weinberg equilibrium test for 18 SNPs obtained in 1,588 cows are presented in Table 1. Except for g.34061171T/C locating on *ZEB1* (*p*-value > 0.002), allele frequencies of all remaining SNPs were not significantly deviated from the Hardy-Weinberg equilibrium. The distributions of SNP clustering (Supplementary Figure S1) suggested that the genotyping qualities were generally good for all 18 SNPs.

From annotation, one SNP (g.34066997C/G, gene *ZEB1*) is in the upstream regulatory region, two (g.33267172C/T, *CACNB2* and g.34063562C/G, *ZEB1*) in exon region, and four in downstream regulatory region (g.32664855G/A, g.32665313G/A and g.32751518G/A in *SLC39A12*, and g.34061171T/C in *ZEB1*). In addition, 11 SNPs were detected from intronic regions, including eight in *CACNB2* (g.33253706A/C, g.33284577T/C, g.33258042G/T, g.33258138T/G, g.33258186G/A, g.33258354A/G, g.33267056T/G, and g.33267296G/C), two in *SLC39A12* (g.32698687A/G and g.32668290G/A), and one in *ZEB1* (g.34110507T/A). The locus g.33258042G/T located in the intron of *CACNB2* is a newly discovered variant.

### 3.2 Association Analyses of SNPs With Reproductive Traits

**FIGURE 2:**
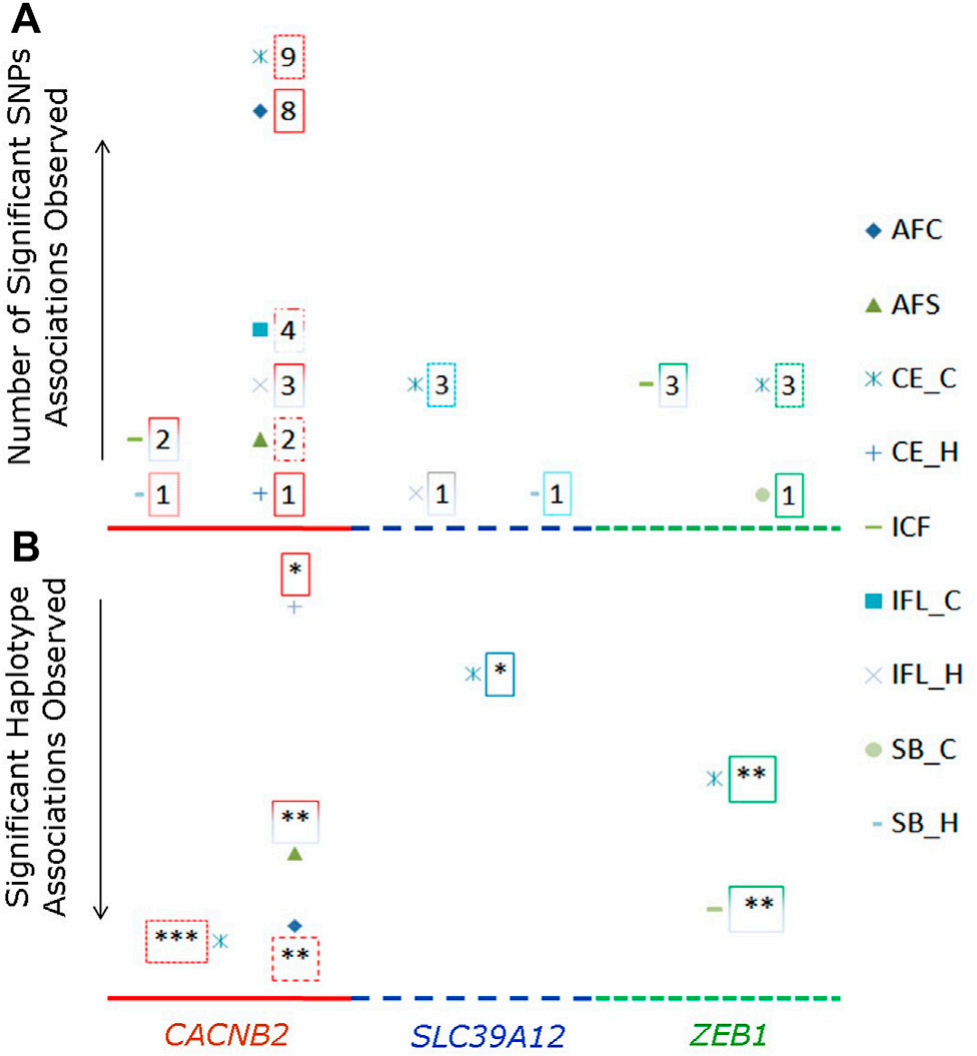
Number of SNPs **(A)** or haplotype blocks **(B)** that were significantly associated with reproductive traits in gene *CACNB2*, *SLC39A12* and *ZEB1*. The legends for nine reproductive traits are shown on the right side. In graph “B”, the “*” represents the significant association on the level of *p* < 0.05, “**” represents *p* < 0.01, and “***” represents *p* < 0.001.

Significant associations with reproductive traits (*p* < 0.05 after Bonferroni correction) were observed in all three candidate genes (Supplementary Table S4). The total number of significant associations detected for each examined SNP or haplotype block is shown in Figure 2A. Most number of significant associations were detected for *CACNB2*, in which nine SNPs were associated with eight reproductive traits. Furthermore, all nine SNPs used in association analysis had significant effects on multiple reproductive traits, with the number of associated reproductive traits ranged from two (g.33267296G/C for CE_C and ICF) to five (g.33258042G/T for AFC, CE_C, CE_H, ICF, and IFL_C, and g.33258186G/A for AFC, AFS, CE_C, IFL_H, and SB_H). When counting by traits, there were nine and eight SNPs in *CACNB2* associated with CE_C and AFC, respectively. However, no SNP within *CACNB2* had significant association with SB_C.

Regarding the gene *SLC39A12*, g.32664855G/A, g.32665313G/A, and g.32751518G/A were significantly associated with CE_C, while g.32664855G/A and g.32751518G/A within *SLC39A12* were significantly associated with both ICF and SB_H. For *ZEB1*, there were four SNPs significantly associated with at least one reproductive trait, including g.34066997C/G and g.34063562C/G for CE_C and ICF, g.34061171T/C for CE_C, CE_H, ICF, and SB_C, and g.34110507T/A for AFC.

The proportions of phenotypic variance explained by each SNP for every reproductive trait are presented in Supplementary Table S5. Among these 18 SNPs, the SNP g.33267056T/G in gene *CACNB2* explained the largest phenotypic variance for AFC (0.011%), CE_C (0.002%), and IFL_C (0.004%), while the SNP g.32751518G/A in gene *SLC39A12* explained the largest variance for SB_H (0.038%). For gene *ZEB1*, g.34061171T/C explained a large phenotypic variance for ICF (0.002%), SB_C (0.004%), and SB_C (0.009%).

### 3.3 Association Analyses of Haplotype Blocks with Reproductive Traits

**FIGURE 3:**
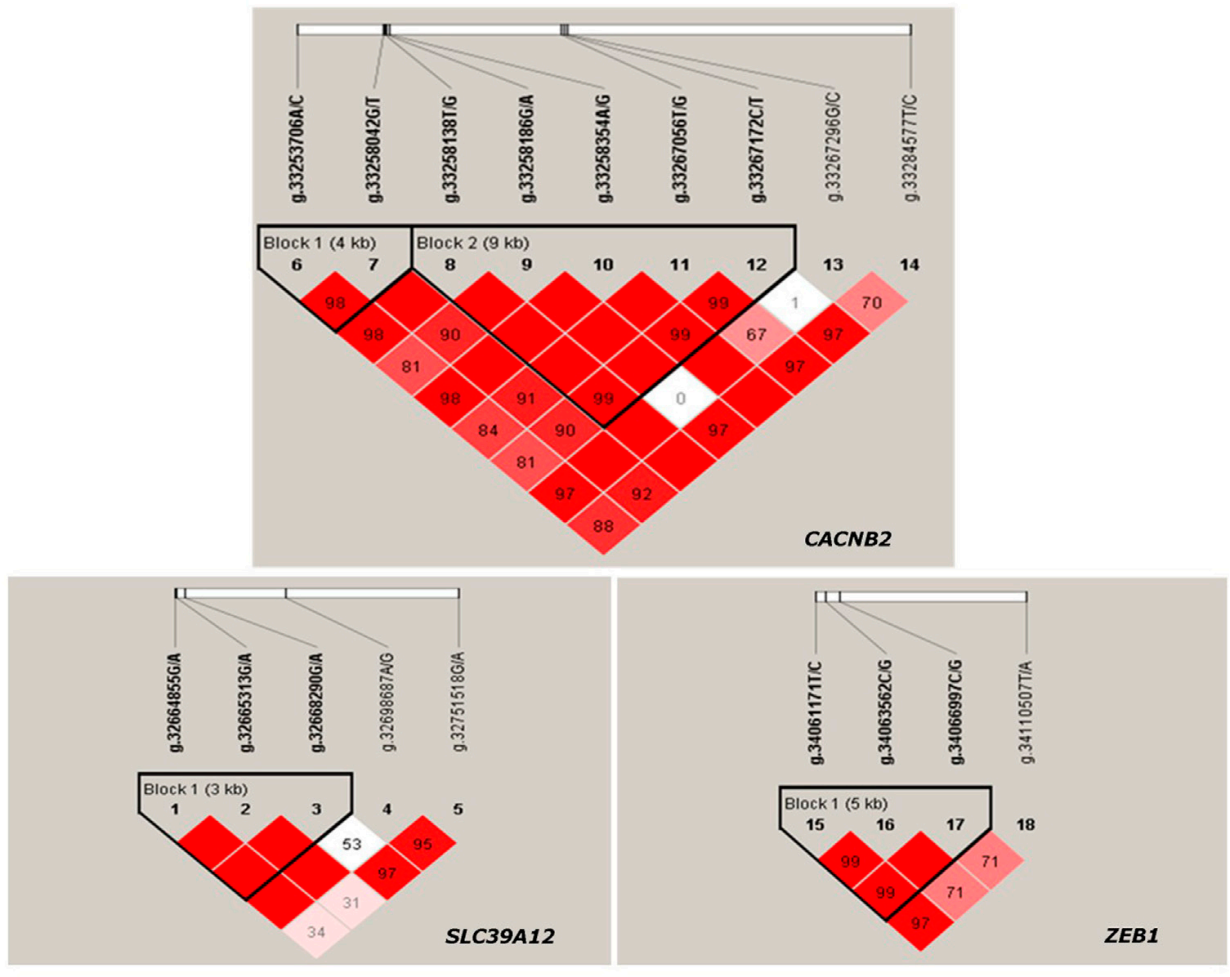
Haplotype blocks constructed based on Linkage disequilibrium (LD) in Chinese Holstein cows for gene *CACNB2*, *SLC39A12* and *ZEB1*.

The LD between every two SNPs were estimated for each candidate gene. Two haplotype blocks (Figure 3) were constructed for *CACNB2*, including three haplotypes for block 1 (consisting of g.33253706A/C and g.33258042G/T) and four haplotypes for block 2 (consisting of g.33258186G/A, g.33258354A/G, g.33267056T/G, and g.33267172C/T). Only one haplotype block (Figure 3) was constructed in *SLC39A12* (three haplotypes, consisting of g.32664855G/A, g.32665313G/A, and g.32668290G/A) and also one for *ZEB1* (three haplotypes, consisting of g.34061171T/C, g.34063562C/G, and g.34066997C/G).

The significant associations detected between haplotype blocks and reproductive traits (*p* < 0.05) are shown in Figure 2B and Table 2. Consistent with the results from SNP-based analyses, the block 1 within *CACNB2* was significantly associated with AFC (*p* < 0.05) and CE_C (*p* < 0.01). The haplotype block 2 in *CACNB2* was significantly associated with AFC (*p* < 0.01), CE_C (*p* < 0.01), AFS (*p* < 0.05), and CE_H (*p* < 0.05). Besides, haplotype block in *ZEB1* was significantly associated with ICF (*p* < 0.01) and CE_C (*p* < 0.05).

### 3.4 Gene Expression Analysis in GCs Isolated from Follicle of Different Developmental Stages

**FIGURE 4:**
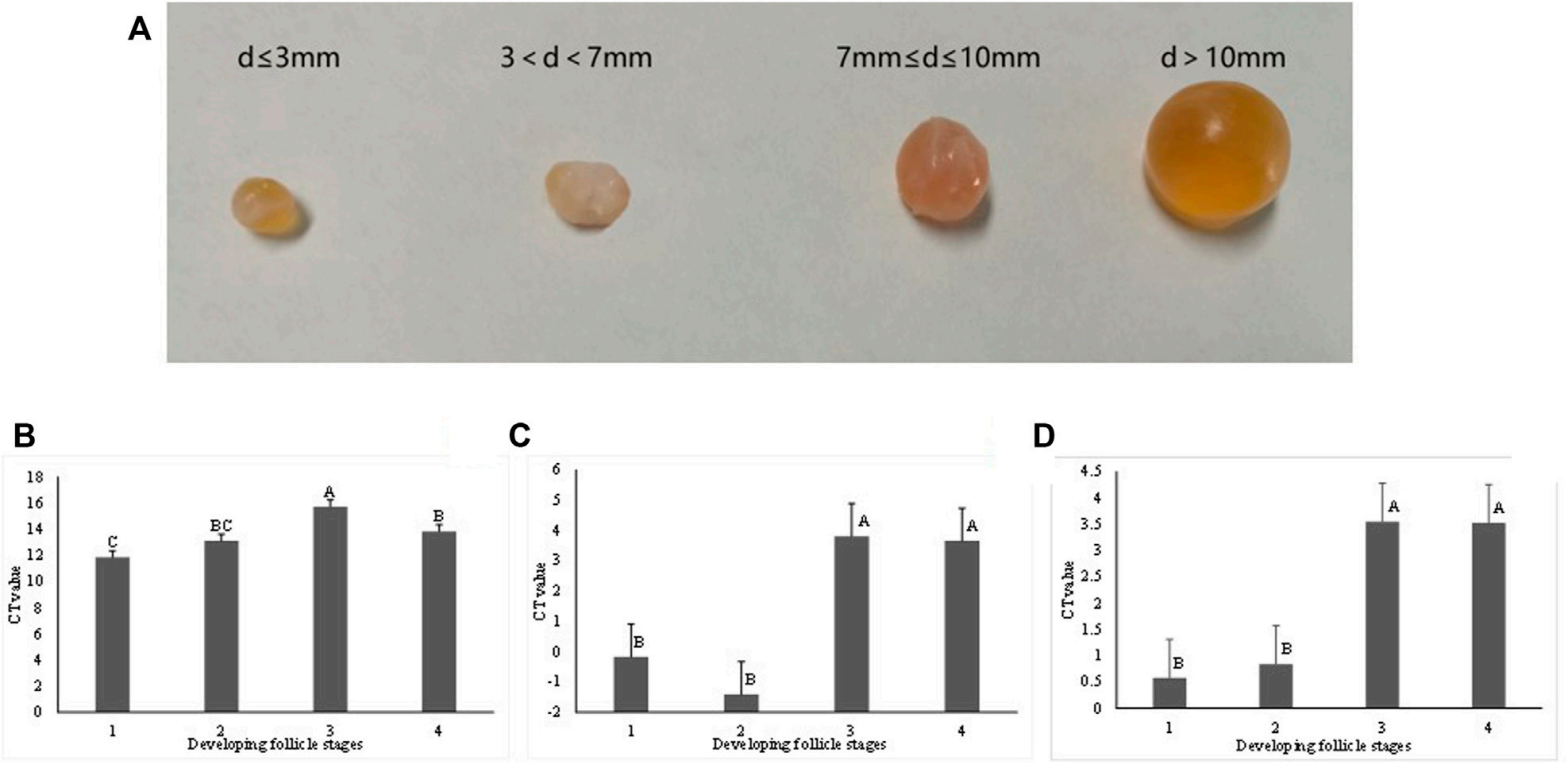
The bovine follicles at different developmental stages **(A)**, and the relative mRNA expression level of the gene *CACNB2* **(B)**, *SLC39A12* **(C)** and *ZEB1* **(D)** in isolated granulosa. Based on the diameter **(D)**, follicles were divided into four stages, including stage 1 (d ≤ 3 mm), stage 2 (3 mm < d ≤ 7 mm), stage 3 (7 mm < d ≤ 10 mm) and stage 4 (d > 10 mm). Different letters are used when the expression levels are significantly different between two stages (*p* < 0.05).

By using qRT-PCR, the relative expression level of gene *CACNB2*, *SLC39A12*, and *ZEB1* was measured in GCs which were isolated from bovine ovarian follicular representing different development stages (Figure 4A). The relative mRNA expression of *CACNB2* in the first stage was significantly higher than that observed in other stages (*p* < 0.05) (Figure 4B). The expression level of the gene *SLC39A12* in GCs was first increased in the early stages and then decreased significantly in later stages (*p* < 0.05) (Figure 4C). There was no significant difference in the relative expression of *ZEB1* gene at follicular development stage 1 and 2 (Figure 4D), but the gene expression level was significantly higher than that from stage 3 and 4. In general, the differential expression level of gene *CACNB2*, *SLC39A12*, and *ZEB1* in GCs at different development stages implied that they might play important roles in reproductive process of cows.

## 4 Discussion

In our previous GWAS of Chinese and Nordic Holstein populations, *CACNB2*, *SLC39A12*, and *ZEB1* were suggested as candidate genes for reproductive traits. This study confirmed the association of genes *CACNB2*, *SLC39A12* and *ZEB1* with reproductive performances in dairy cattle.

The SNP g.33258186G/A locating in the intron region of *CACNB2* was observed to have significant associations with all heifer traits (AFC, AFS, IFL_H, SB_H, and CE_H), with the genotype GG as the advantageous genotype. Similarly, another *CACNB2* SNP (g.33267172C/T) in the exon region was significantly associated with AFC, IFL_H, CE_C, and AFS and explained large phenotypic variations in heifer traits. Although not reaching significant threshold, the advantageous genotype CC of g.33267172C/T also has favorable effects on SB_H and CE_H. All these findings suggested the critical roles of g.33258186G/A and g.33267172C/T in heifer reproduction. The relationship between *SLC39A12* and reproductive performance of dairy cattle have been less reported previously. Amongst nine traits studied in this study, *SLC39A12* was mostly responsive for SB_H. In *SLC39A12*, the SNP g.32751518G/A located in the 3′UTR region (related to post-transcriptional gene expression and affecting the regulation of mRNA expression) was significant associated with both CE_C and SB_H. Further studies are necessary to understand its biological relevance (Edwards et al., 2013; Sammeth et al., 2008). Interestingly, for two SNPs (g.32665313G/A (significantly associated with CE_C) and g.32668290G/A) which were significantly deviated from HWE, only a few individuals (2 and 13, respectively) carrying the rare genotypes. This together with the reported association of *SLC39A12* with bovine hereditary zinc-deficiency (BHZD) syndrome (Yuzbasiyan-Gurkan and Bartlett, 2006), suggesting that there could be lethal alleles located in *SLC39A12*. Previous GWAS also reported strong associations with fertility traits in a region containing *ZEB1* along with other genes on BTA13 (Höglund et al., 2009; Höglund et al., 2014; Liu et al., 2017b). In this study, we did observe significant associations for *ZEB1*, including SNPs g.34066997C/G and g.34063562C/G (both located in the exon region) for ICF and CE_C.

The mammalian ovarian follicle consists of one oocyte that undergoes a series of biological events, including ovulation, fertilization, and formation of an embryo. Oocyte is surrounded by granulosa and theca cells, which can produce signals and hormones to enable the development of oocyte (Su et al., 2004). During the follicle development, GCs replicate, secrete hormones, and provide a critical microenvironment for follicular growth (Petro et al., 2012). Proliferation and differentiation of GCs are essential for normal follicular growth, oocyte development, ovulation, and luteinization (Eppig, 2001; Bettaieb and Averill-Bates, 2008). In this study, the expressions of *CACNB2*, *SLC39A12,* and *ZEB1* were quantified in GCs from different ovarian follicle developmental stages in mature cows. For example, the relative expression of *CACNB2* was at its highest level at the beginning of follicular development and then drastically attenuated at later stages.

In Sahiwal cattle (Vineeth et al., 2020), Japanese Holstein (Sugimoto et al., 2013), and Nordic Holstein (Höglund et al., 2014), the gene *CACNB2* has been reported to be the candidate gene for reproductive traits. In a meta-analysis of heifer reproductive traits, serval genes involved in the calcium signaling pathway, including *CACNB2*, was identified as the candidate genes (Tahir et al., 2021). The *CACNB2* is a member of the voltage-gated calcium channel superfamily. Calcium regulation, through voltage-gated channels, is a prerequisite for GnRH-induced activation of extracellular signal-regulated kinase, which in turn leads to the secretion of FSH (Bliss et al., 2012; Dang et al., 2014). Knockdown of *CACNB2* can reduce FSH secretion, while the overexpression of *CACNB2* can increase its release (Sugimoto et al., 2013). There is also evidence that showing *CACNB2* can control for the secretion of FSH. Following our previous GWAS in Chinese and Nordic Holstein population, the present study further confirmed the genetic effect on reproductive traits in cattle, at both SNP and mRNA expression levels.

Zinc is important for oocyte meiotic progression (Kim et al., 2010), of which the deficiency of zinc during pregnancy can lead to many pregnancy complications in human, such as early embryo death, intrauterine growth retardation and teratogenesis, or miscarriage (Uriu-Adams et al., 2010). The SLC39 family, including *SLC39A12*, is mainly involved in the transport of zinc into the cytoplasm (Kong et al., 2014), *via* secreting a kind of protein with a structure characteristic of zinc transporter. In the cumulus-oocyte complex, the removal of cumulus granulosa cells leads to an increase in zinc content in oocytes (Lisle et al., 2013). Our results showed that the expression of *SLC39A12* was different across different follicle stages. Based on these observations, our hypothesis is that in cattle, *SLC39A12* could contribute to oocyte maturation by involving in the process of zinc homeostasis and have the same capacity as reported in humans. Further studies towards understanding zinc metabolism needs to be done in order to discover the mechanism of the action of *SLC39A12* gene at the junction of GCs and oocyte.

The gene *ZEB1* (an alternative name of *AREB6*) was reported to play a role in the expression of epithelial-mesenchymal transition (Yamakoshi et al., 2012), which is partly required during the process of vegetative ectoderm junction. It was found that miR-200b, miR-429, and their target gene (*ZEB1*), act in the regulation of mammalian reproduction. Eliminating these miRNAs inhibits luteinizing hormone (LH) synthesis by repressing transcription of its b-subunit gene, which can cause lowered serum LH concentration, an impaired LH surge, and failure to ovulate. The hypothalamo-pituitary-ovarian axis was shown to require miR-200b and miR-429 to support ovulation (Hasuwa et al., 2013). In this study, differential expression with varying follicular developmental stages indicated that *ZEB1* was likely to be involved in the process of follicular development. In bovine, it has been reported that *ZEB1* was a candidate gene for temperament in Brahman and Yunling cattle (Chen et al., 2020), residual feed intake in Angus (Weber et al., 2016), and growth and meat quality traits in Brazilian Nellore (Mudadu et al., 2016). To our knowledge, no literature ever linked it to reproductive traits except for our previous GWAS in Chinese and Nordic Holsteins (Liu et al., 2017b).

## 5 Conclusion

Our previous GWAS identified genes *CACNB2*, *SLC39A12*, and *ZEB1* as promising candidates for reproduction traits in dairy cattle. In this study, we further validated effects of these genes on reproductive traits by association analyses at a population level and mRNA expression assay at a cell level. Numerous SNPs within *CACNB2*, *SLC39A12*, and *ZEB1* were significantly associated with reproductive traits, such as g.33258186G/A and g.33267172C/T within *CACNB2*, g.32751518G/A within *SLC39A12*, and g.34066997C/G and g.34063562C/G within *ZEB1*. A novel SNP g.33258042G/T located in the intron of CACNB2 was discovered. There were significant differences on the relative expressions of *CACNB2*, *SLC39A12,* and *ZEB1* in granulosa cell at the different developmental stage of ovarian follicle. Findings from this study would contribute towards genetic selection of reproductive traits by improving the reliability of genomic evaluation in dairy cattle.

## Data Availability

The data presented in the study are deposited in the figshare repository, accession number 10.6084/m9.figshare.19846765.

## Abbreviations

AFS, age at the first service; AFC, age at the first calving; *CACNB2*, calcium voltage-gated channel auxiliary subunit beta 2; CE_C, calving ease in cows; CE_H, calving ease in heifers; ICF, the interval from calving to the first insemination; IFL_C, the interval from the first to last insemination in cows; IFL_H, the interval from the first to last insemination in heifers; SB_C, stillbirth in cows; SB_H, stillbirth in heifers; *SLC39A12*, solute carrier family 39 member 12; *ZEB1*, zinc finger E-box binding homeobox 1.
