# Supplementary material for: A Post-GWAS Functional Analysis Confirming Effects of Three BTA13 Genes CACNB2, SLC39A12, and ZEB1 on Dairy Cattle Reproduction
Source: Front Genet. 2022 Jun 8;13:882951. doi: 10.3389/fgene.2022.882951 (PMC9216173; doi:10.3389/fgene.2022.882951)
Supplement: Supplementary file 1 [file Table1.DOCX]

**Table S1.** Primers (50) of the gene *CACNB2*, *SLC39A12* and *ZEB1* for pooled DNA sequencing.

| **Primers of *CACNB2*** | | | | | | | | | |
| --- | --- | --- | --- | --- | --- | --- | --- | --- | --- |
| Primer | Location | Primer Sequence (5’-3’) | Product length (bp) | Annealing Temp. (oC) | Primer | Location | Primer Sequence (5’-3’) | Product length (bp) | Annealing Temp. (oC) |
| **C-5'UTR1 F’. R’** | 5’ Regulatory region | CTTATTGGGTTTCTCGTATC | 381 | 56 | **C-E8 F’. R’** | exon 8 | TTTGTTGCCTTGGAGATG | 588 | 56 |
|  |  | TGTTGCCTCTGTATCACTTT |  |  |  |  | CAGGTGTAGGAACGGAGA |  |  |
| **C-5'UTR2+E1 F’. R’** | 5’ Regulatory region | AGCACGTACTGCAAGACGA | 838 | 56 | **C-E9 F’ R** | exon 9 | CCTGGAGAATGGAAACG | 619 | 56 |
|  |  | CACCAGACAGACACGGATAG |  |  |  |  | TGTCAAAGCGAGGAGT |  |  |
| **C-E2 F’. R’** | exon 2 | GCTAACTGCCGTGTCA | 377 | 56 | **C-E10 F’. R’** | exon 10 | CTAGGCTTGAGTGGTCAGA | 797 | 56 |
|  |  | ACTGCCTTCTTCTACCAA |  |  |  |  | GCCAGTGGAAGAGGAGG |  |  |
| **C-E3 F’. R’** | exon 3 | AGGTTTCCCCTGTTG | 498 | 56 | **C-E11 F’. R’** | exon 11 | AAAGCGTGAATAATGAACC | 292 | 56 |
|  |  | GGAGCCTTGAGGTTAT |  |  |  |  | AGAAGCCCACTGCTAATG |  |  |
| **C-E4 F’. R’** | exon 4 | GACGGATTGTTGAGCAT | 555 | 56.5 | **C-E12 F’. R’** | exon 12 | TACCCGAGGAGGACCAA | 523 | 59 |
|  |  | CTATTTGACTTATTTGTGGG |  |  |  |  | AGGAAGAAACCCATACAACC |  |  |
| **C-E5 F’. R’** | exon 5 | GTCTATTTGCGATCCTGG | 496 | 56 | **C-E13-1 F’. R’** | exon 13 | AAAAGGCTGAAATGAAATCTAAC | 691 | 58 |
|  |  | TCATTTCTGCTCCCTCTG |  |  |  |  | CTAAAAGGCTGAAATGAAATCTA |  |  |
| **C-E6 F’. R’** | exon 6 | TGAGCGTGAAACCAATG | 594 | 56 | **C-E13-2 F’. R’** | exon 13 | GGCGGTGAGCACAGACA | 596 | 59 |
|  |  | GTGGGACTGAATGCTAATG |  |  |  |  | CGGCAAGAATCAGGAAAC |  |  |
| **C-E7 F’. R’** | exon 7 | CTTGGGTTGGGTTTATTTAG | 510 | 58 | **C-3'UTR F’. R’** | 3’ Regulatory region | TGCGAGCAAGTGGAGG | 647 | 60 |
|  |  | CACTCTTGGAGGGACTG |  |  |  |  | GAGGCAGAGCAGGTTTCA |  |  |
| **Primers of *SLC39A12*** | | | | | | | | | |
| **S-5'UTR-1 F’. R’** | 5’ Regulatory region | AAGGTTGATTTCAGGGTTG | 551 | 54 | **S-E6 F’. R’** | exon 6 | TGCTTAGGCTGCTGGTT | 729 | 56 |
|  |  | TTGATTGGCTATGAAGTGGT |  |  |  |  | GTGTCTTTGGGCAAGTTAT |  |  |
| **S-5'UTR-2 F’. R’** | 5’ Regulatory region | GGGAACCTGATGATTGGA | 662 | 54 | **S-E7 F’. R’** | exon 7 | GTTCCCTGACCAGCAAT | 825 | 54 |
|  |  | AGAGGGATTTCTGAGTTGG |  |  |  |  | CCAGAAATGACACCCAAT |  |  |
| **S-5'UTR-3 F’. R’** | 5’ Regulatory region | CGGTGGCTGACTTAGGTATG | 582 | 59 | **S-E8 F’. R’** | exon 8 | AGTGGAAGGATTCATACG | 516 | 56 |
|  |  | TCATTTGTAGGCTCTGTCCC |  |  |  |  | GGGCCAGCAACAGAT |  |  |
| **S-5'UTR-4 F’. R’** | 5’ Regulatory region | GTCCCTGAGACTGAGATGG | 511 | 59 | **S-E9 F’. R’** | exon 9 | CTCGGCTCTACCTTC | 461 | 56.6 |
|  |  | GGATTTCTGAGTTGGGTTCT |  |  |  |  | AAGAATGGAGTAAAGTTG |  |  |
| **S-5'UTR-5+E1 F’. R’** | 5’ Regulatory region | TAGCGACACCGATTTG | 647 | 50 | **S-E10 F’. R’** | exon 10 | AGCAGCGACCATACCT | 574 | 58 |
|  |  | AAAGGAAAGGACGAAAA |  |  |  |  | TTCTGGCTCCAAGAAAT |  |  |
| **S-E2 F’. R’** | exon 2 | TTGAAAGTCTAAACCCACCAT | 885 | 54 | **S-E12 F’. R’** | exon 12 | GTTTTAGAATTTTGGTGAG | 698 | 52 |
|  |  | AAGCAGCATCTATCTAACCAA |  |  |  |  | ACTTAGTTTGCTTATGGT |  |  |
| **S-E3 F’. R’** | exon 3 | CCCTGACTGTCTTACTTGA | 704 | 53 | **S-3'UTR1- F’. R’** | 3’ Regulatory region | TCGTGTCACTCAGTCACATA | 554 | 56 |
|  |  | TCAGAATAGAACTGCCTTG |  |  |  |  | AGCCCCACTCAGACTTAC |  |  |
| **S-E4 F’. R’** | exon 4 | CTGTGATGCCCAAGTTTAG | 479 | 55 | **S-3'UTR2- F’. R’** | 3’ Regulatory region | GGGCAGGCAGTTCTTC | 645 | 55 |
|  |  | CCACAATGCTTCCACCT |  |  |  |  | GGTTGAGGTTAGGCTTGAT |  |  |
| **S-E5 F’. R’** | exon 5 | TAGGCAAGATGTTGTAAGG | 606 | 50 | **S-3'UTR3- F’. R’** | 3’ Regulatory region | ATCCATCTCGGGAGTTAC | 901 | 59 |
|  |  | TTCAGTATGGAGGAAAGAG |  |  |  |  | TTAGGCTATTGGGTTGC |  |  |
| **Primers of *ZEB1*** | | | | | | | | | |
| **Z-5'UTR-1 F’. R’** | 5’ Regulatory region | CGCACAGCAAAGAATAGTTAC | 537 | 54 | **Z-E7 F’. R’** | exon 7 | CTGCTACGATTTGAGG | 473 | 52 |
|  |  | AGATACCAGCACAAATACCAAG |  |  |  |  | ACATCTTGTAAAACGCTA |  |  |
| **Z-5'UTR-2 F’. R’** | 5’ Regulatory region | ATTTGTTCAGCACCTACTGTGA | 649 | 55 | **Z-E8-1 F’. R’** | exon 8 | GTACTTAAAGTGGCGGTAGATG | 624 | 53 |
|  |  | CCGAAAGAAGGGCGAGA |  |  |  |  | CAGTGGTAGGTTCACGGAAT |  |  |
| **Z-5'UTR-3 F’. R’** | 5’ Regulatory region | AGGTGGCTCACTGGTAAA | 560 | 65 | **Z-E8-2 F’. R’** | exon 8 | CGGAGATGGCAGTTTGTC | 698 | 53 |
|  |  | GCTCGGTTCAAGTGGTAAT |  |  |  |  | GGGCAGTGACGGTAGGTAT |  |  |
| **Z-E2 F’. R’** | exon 2 | GCAGTAGGCAGTAGATAGG | 833 | 56 | **Z-E8-3 F’. R’** | exon 8 | TATTGGTGCTTGACATTTAT | 491 | 60.6 |
|  |  | ATCAGTGGAGGCATTAGA |  |  |  |  | TCTGTAGAGGGGCTGAA |  |  |
| **Z-E3 F’. R’** | exon 3 | TAAGCAAACTAAAGCCAAAG | 895 | 56 | **Z-E9 F’. R’** | exon 9 | CAGCGTCCTTTCTTTCGG | 627 | 55 |
|  |  | GTGAGGTAAAGCCCAGAA |  |  |  |  | GCACTTGTCGCACTGGTAG |  |  |
| **Z-E4 F’** | exon 4 | TTCACTCATTTTGCTTCTC | 436 | 60.6 | **Z-E10 F’. R’** | exon 10 | GAACGACTCCGATTCCACG | 819 | 56 |
|  |  | TCATACAGTGCCCCAT |  |  |  |  | GCTGCCCTCACTGACTTTG |  |  |
| **Z-E5 F’. R’** | exon 5 | CTAAGGATTCAGCCAACC | 882 | 61 | **Z-3'UTR-1 F’. R’** | 3’ Regulatory region | CCAGCCTCCGTTCAT | 791 | 55 |
|  |  | GACGACCAACATAGATACAG |  |  |  |  | AAACCGCAGAGCATC |  |  |
| **Z-E6 F’. R’** | exon 6 | TTTTCTGGGCATCACG | 625 | 56 | **Z-3'UTR-2 F’. R’** | 3’ Regulatory region | AGGAAGCCAAATTAGGATAA | 485 | 50 |
|  |  | ATAAATGAGTTGAAAGGGAC |  |  |  |  | AGGAAAGACTGTGAAACCC |  |  |
