# Supplementary material for: A Post-GWAS Functional Analysis Confirming Effects of Three BTA13 Genes CACNB2, SLC39A12, and ZEB1 on Dairy Cattle Reproduction
Source: Front Genet. 2022 Jun 8;13:882951. doi: 10.3389/fgene.2022.882951 (PMC9216173; doi:10.3389/fgene.2022.882951)
Supplement: Supplementary file 2 [file Table2.DOCX]

**Table S2.** The descriptive statistics of EBV and variance components for 9 reproductive traits in 1,588 Chinese Holstein cows.

|  | EBV | | | | Variance Components | | |
| --- | --- | --- | --- | --- | --- | --- | --- |
| Trait^1^ | Mean | SD | Min | Max | $\sigma_{a}^{2}$ | $\sigma_{pe}^{2}$ | $\sigma_{e}^{2}$ |
| AFS | -14.72 | 10.54 | -64.43 | 65.80 | 795.13 | - | 2,172.21 |
| AFC | -8.75 | 5.56 | -25.73 | 18.00 | 402.44 | - | 4,486.82 |
| CE_C | 5.97 | 6.40 | -9.57 | 42.71 | 0.0005 | 0.0011 | 0.0547 |
| CE_H | 0.026 | 0.033 | -0.154 | 0.169 | 0.0022 | - | 0.0626 |
| ICF | 0.008 | 0.013 | -0.035 | 0.072 | 14.98 | 18.07 | 450.98 |
| IFL_C | 2.15 | 5.27 | -13.47 | 25.60 | 117.45 | 369.66 | 6,074.61 |
| IFL_H | 0.10 | 1.06 | -4.07 | 4.50 | 330.95 | - | 3,287.89 |
| SB_C | 0.016 | 0.015 | -0.108 | 0.079 | 0.0023 | 0.0008 | 0.0195 |
| SB_H | 0.001 | 0.006 | -0.014 | 0.027 | 0.0074 | - | 0.0228 |

^1^ AFS, age at the first service; AFC, age at the first calving; CE_C, calving ease in cows; CE_H, calving ease in heifers; ICF, the interval from calving to the first insemination; IFL_C, the interval from the first to last insemination in cows; IFL_H, the interval from the first to last insemination in heifers; SB_C, stillbirth in cows; SB_H, stillbirth in heifers.
