# Supplementary material for: A Post-GWAS Functional Analysis Confirming Effects of Three BTA13 Genes CACNB2, SLC39A12, and ZEB1 on Dairy Cattle Reproduction
Source: Front Genet. 2022 Jun 8;13:882951. doi: 10.3389/fgene.2022.882951 (PMC9216173; doi:10.3389/fgene.2022.882951)
Supplement: Supplementary file 3 [file Table5.DOCX]

**Table S4:** Association of SNPs within gene *CACNB2*, *SLC39A12*, and *ZEB1* with reproductive traits in Holstein cows (Least square mean ± Standard error).

| SNP | Genotype | AFC^1^ | AFS | CE_C | CE_H | ICF | IFL_C | IFL_H | SB_C | SB_H |
| --- | --- | --- | --- | --- | --- | --- | --- | --- | --- | --- |
| ***CACNB2*** | GG(430) | -10.138±0.308^b^ | -17.878±0.374 | 0.002±0.000^a^ | 0.008±0.001 | 0.494±0.054 | 2.030±0.246 | 6.380±0.302 | 0.014±0.001 | 0.029±0.002 |
| g.33258042G/T | TG(740) | -8.840±0.235^a^ | -17.324±0.285 | 0.000±0.000^b^ | 0.008±0.000 | 0.374±0.041 | 2.661±0.187 | 6.795±0.231 | 0.015±0.000 | 0.029±0.001 |
|  | TT(295) | -8.874±0.373^a^ | -17.291±0.452 | 0.000±0.000^b^ | 0.010±0.001 | 0.292±0.065 | 2.926±0.297 | 6.874±0.366 | 0.015±0.001 | 0.026±0.002 |
|  |  | 0.002 | 0.447 | <.0001 | 0.071 | 0.048 | 0.041 | 0.471 | 0.344 | 0.253 |
| ***CACNB2*** | GG(316) | -8.632±0.359^a^ | -17.386±0.435 | 0.000±0.000^b^ | 0.009±0.001 | 0.293±0.063 | 2.964±0.287^a^ | 7.075±0.353 | 0.016±0.001 | 0.027±0.002 |
| g.33258138T/G | GT(744) | -8.971±0.234^a^ | -17.239±0.283 | 0.000±0.000^b^ | 0.008±0.000 | 0.375±0.041 | 2.627±0.187^ab^ | 6.674±0.230 | 0.015±0.000 | 0.029±0.001 |
|  | TT(400) | -10.101±0.319^b^ | -18.000±0.386 | 0.002±0.000^a^ | 0.009±0.001 | 0.491±0.056 | 1.998±0.255^b^ | 6.432±0.313 | 0.014±0.001 | 0.028±0.002 |
|  |  | 0.003 | 0.276 | <.0001 | 0.225 | 0.059 | 0.032 | 0.392 | 0.228 | 0.793 |
| ***CACNB2*** | AA(147) | -9.200±0.527^ab^ | -18.126±0.640^ab^ | 0.000±0.000^b^ | 0.010±0.001 | 0.387±0.093 | 2.774±0.423 | 7.444±0.519^ab^ | 0.016±0.001 | 0.035±0.003^a^ |
| g.33258186G/A | AG(629) | -8.411±0.254^a^ | -16.845±0.308^a^ | 0.000±0.000^b^ | 0.008±0.000 | 0.369±0.045 | 2.619±0.204 | 7.249±0.250^a^ | 0.014±0.001 | 0.027±0.001^b^ |
|  | GG(653) | -9.901±0.249^b^ | -17.885±0.302^b^ | 0.001±0.000^a^ | 0.008±0.000 | 0.414±0.044 | 2.300±0.200 | 6.073±0.245^b^ | 0.015±0.001 | 0.028±0.001^b^ |
|  |  | 0 | 0.03 | <.0001 | 0.119 | 0.779 | 0.418 | 0.001 | 0.637 | 0.02 |
| ***CACNB2*** | AA(407) | -10.153±0.316^b^ | -18.030±0.383 | 0.002±0.000a | 0.009±0.001 | 0.489±0.056 | 1.988±0.252^b^ | 6.410±0.310 | 0.014±0.001 | 0.029±0.002 |
| g.33258354A/G | GA(739) | -8.995±0.234^a^ | -17.238±0.285 | 0.000±0.000b | 0.008±0.000 | 0.381±0.041 | 2.602±0.187^ab^ | 6.685±0.230 | 0.015±0.000 | 0.029±0.001 |
|  | GG(319) | -8.697±0.357^a^ | -17.252±0.433 | 0.000±0.000b | 0.009±0.001 | 0.304±0.063 | 3.034±0.285^a^ | 7.011±0.351 | 0.016±0.001 | 0.027±0.002 |
|  |  | 0.003 | 0.218 | <.0001 | 0.247 | 0.079 | 0.02 | 0.439 | 0.252 | 0.749 |
| ***CACNB2*** | GG(224) | -8.166±0.427^a^ | -17.480±0.532 | -0.001±0.000^c^ | 0.009±0.001 | 0.332±0.074 | 3.302±0.336^a^ | 7.494±0.414^a^ | 0.016±0.001 | 0.029±0.002 |
| g.33267056T/G | GT(591) | -8.889±0.263^a^ | -17.192±0.327 | 0.000±0.000^b^ | 0.008±0.001 | 0.392±0.045 | 2.838±0.207^a^ | 6.941±0.255^ab^ | 0.015±0.001 | 0.027±0.001 |
|  | TT(376) | -10.143±0.329^b^ | -18.134±0.410 | 0.002±0.000^a^ | 0.009±0.001 | 0.528±0.057 | 2.015±0.259^b^ | 6.173±0.319^b^ | 0.015±0.001 | 0.028±0.002 |
|  |  | 0.001 | 0.198 | <.0001 | 0.175 | 0.069 | 0.005 | 0.031 | 0.638 | 0.771 |
| ***CACNB2*** | CC(657) | -9.922±0.248^b^ | -17.928±0.301^b^ | 0.001±0.000^a^ | 0.008±0.000 | 0.407±0.044 | 2.324±0.200 | 6.132±0.244^b^ | 0.015±0.001 | 0.028±0.001 |
| g.33267172C/T | TC(620) | -8.464±0.256^a^ | -16.844±0.310^a^ | 0.000±0.000^b^ | 0.008±0.001 | 0.374±0.046 | 2.595±0.206 | 7.156±0.252^a^ | 0.015±0.001 | 0.027±0.001 |
|  | TT(153) | -9.186±0.517^ab^ | -17.899±0.626^ab^ | 0.000±0.000^b^ | 0.010±0.001 | 0.381±0.092 | 2.967±0.416 | 7.261±0.508^ab^ | 0.016±0.001 | 0.034±0.003 |
|  |  | 0 | 0.033 | <.0001 | 0.197 | 0.871 | 0.328 | 0.007 | 0.694 | 0.066 |
| ***CACNB2*** | CC(64) | -8.200±0.801 | -16.122±0.970 | 0.000±0.001^ab^ | 0.011±0.002 | 0.034±0.141^b^ | 2.907±0.637 | 7.008±0.785 | 0.014±0.002 | 0.024±0.004 |
| g.33267296G/C | CG(491) | -8.984±0.290 | -17.876±0.351 | 0.000±0.000^b^ | 0.008±0.001 | 0.365±0.051^ab^ | 2.902±0.230 | 6.694±0.284 | 0.016±0.001 | 0.027±0.001 |
|  | GG(897) | -9.436±0.214 | -17.347±0.259 | 0.001±0.000^a^ | 0.009±0.000 | 0.428±0.038^a^ | 2.291±0.170 | 6.656±0.210 | 0.014±0.000 | 0.029±0.001 |
|  |  | 0.193 | 0.174 | 0.023 | 0.085 | 0.022 | 0.085 | 0.91 | 0.074 | 0.265 |
| ***CACNB2*** | AA(462) | -9.955±0.296^b^ | -17.655±0.359 | 0.002±0.000^a^ | 0.009±0.001^ab^ | 0.485±0.052 | 2.018±0.236 | 6.390±0.290 | 0.014±0.001 | 0.028±0.001 |
| g.33253706A/C | CA(736) | -8.911±0.234^a^ | -17.421±0.284 | 0.000±0.000^b^ | 0.008±0.000^b^ | 0.367±0.041 | 2.642±0.187 | 6.737±0.230 | 0.015±0.000 | 0.029±0.001 |
|  | CC(287) | -8.761±0.376^a^ | -17.131±0.456 | 0.000±0.000^b^ | 0.010±0.001^a^ | 0.296±0.066 | 2.867±0.300 | 6.918±0.369 | 0.015±0.001 | 0.025±0.002 |
|  |  | 0.009 | 0.663 | <.0001 | 0.042 | 0.058 | 0.045 | 0.481 | 0.318 | 0.164 |
| ***CACNB2*** | CC(267) | -8.771±0.393^a^ | -17.902±0.474 | -0.001±0.000^c^ | 0.009±0.001 | 0.278±0.069^b^ | 2.931±0.312^a^ | 7.146±0.384 | 0.016±0.001 | 0.028±0.002 |
| g.33284577T/C | CT(740) | -8.988±0.236^a^ | -17.192±0.284 | 0.000±0.000^b^ | 0.008±0.000 | 0.351±0.041^ab^ | 2.730±0.187^a^ | 6.712±0.230 | 0.015±0.000 | 0.028±0.001 |
|  | TT(468) | -9.982±0.296^b^ | -17.869±0.357 | 0.002±0.000^a^ | 0.009±0.001 | 0.497±0.052^a^ | 1.956±0.235^b^ | 6.348±0.289 | 0.014±0.001 | 0.028±0.001 |
|  |  | 0.012 | 0.231 | <.0001 | 0.5 | 0.021 | 0.013 | 0.246 | 0.277 | 0.944 |
| ***SLC39A12*** | AA(55) | -9.581±0.865 | -16.965±1.045 | 0.000±0.001^ab^ | 0.010±0.002 | 0.241±0.152 | 2.454±0.690 | 6.201±0.845 | 0.013±0.002 | 0.024±0.004 |
| g.32664855G/A | AG(448) | -8.758±0.303 | -17.879±0.366 | 0.000±0.000^b^ | 0.008±0.001 | 0.297±0.053 | 2.645±0.242 | 6.538±0.296 | 0.015±0.001 | 0.028±0.001 |
|  | GG(959) | -9.408±0.207 | -17.314±0.250 | 0.001±0.000^a^ | 0.009±0.000 | 0.442±0.036 | 2.497±0.165 | 6.753±0.203 | 0.015±0.000 | 0.029±0.001 |
|  |  | 0.191 | 0.393 | 0.013 | 0.131 | 0.049 | 0.874 | 0.715 | 0.589 | 0.521 |
| ***SLC39A12*** | AA(931) | -9.359±0.210 | -17.338±0.252 | 0.001±0.000^a^ | 0.009±0.000 | 0.440±0.037 | 2.470±0.167 | 6.793±0.206 | 0.015±0.000 | 0.029±0.001 |
| g.32665313G/A | AG(461) | -8.856±0.298 | -17.935±0.358 | 0.000±0.000^b^ | 0.008±0.001 | 0.299±0.052 | 2.622±0.237 | 6.468±0.292 | 0.015±0.001 | 0.028±0.001 |
|  | GG(58) | -9.478±0.841 | -16.426±1.010 | 0.000±0.001^ab^ | 0.010±0.002 | 0.259±0.148 | 2.624±0.668 | 5.996±0.824 | 0.013±0.002 | 0.021±0.004 |
|  |  | 0.367 | 0.222 | 0.013 | 0.148 | 0.062 | 0.861 | 0.473 | 0.626 | 0.247 |
| ***SLC39A12*** | AA(13) | -9.706±1.777 | -15.502±2.144 | 0.002±0.002 | 0.011±0.003 | 0.165±0.310 | 1.601±1.412 | 5.489±1.727 | 0.015±0.004 | 0.028±0.009 |
| g.32668290G/A | AG(289) | -8.982±0.378 | -16.955±0.456 | 0.001±0.000 | 0.009±0.001 | 0.414±0.066 | 2.666±0.300 | 6.613±0.367 | 0.015±0.001 | 0.027±0.002 |
|  | GG(1176) | -9.307±0.187 | -17.648±0.226 | 0.001±0.000 | 0.008±0.000 | 0.385±0.033 | 2.502±0.149 | 6.671±0.182 | 0.015±0.000 | 0.028±0.001 |
|  |  | 0.717 | 0.256 | 0.72 | 0.386 | 0.711 | 0.714 | 0.789 | 0.931 | 0.783 |
| ***SLC39A12*** | AA(2) | -11.454±4.526 | -18.898±5.455 | 0.000±0.004 | 0.005±0.009 | 0.108±0.796 | 2.577±3.594 | 11.150±4.406^ab^ | 0.033±0.009 | 0.037±0.022 |
| g.32698687A/G | GA(164) | -8.877±0.501 | -18.118±0.604 | 0.000±0.000 | 0.010±0.001 | 0.493±0.088 | 3.211±0.398 | 7.843±0.488^a^ | 0.014±0.001 | 0.029±0.002 |
|  | GG(1314) | -9.292±0.177 | -17.422±0.213 | 0.001±0.000 | 0.008±0.000 | 0.375±0.031 | 2.434±0.140 | 6.510±0.172^b^ | 0.015±0.000 | 0.028±0.001 |
|  |  | 0.655 | 0.537 | 0.1 | 0.125 | 0.428 | 0.184 | 0.0217 | 0.065 | 0.835 |
| ***SLC39A12*** | AA(225) | -8.624±0.426 | -17.184±0.516 | 0.000±0.000b | 0.010±0.001 | 0.271±0.075 | 2.920±0.340 | 6.723±0.418 | 0.013±0.001 | 0.022±0.002^b^ |
| g.32751518G/A | AG(702) | -9.368±0.242 | -17.833±0.292 | 0.000±0.000b | 0.008±0.000 | 0.389±0.043 | 2.662±0.193 | 6.646±0.237 | 0.015±0.000 | 0.028±0.001^ab^ |
|  | GG(537) | -9.322±0.276 | -17.289±0.334 | 0.001±0.000a | 0.008±0.001 | 0.439±0.049 | 2.158±0.220 | 6.746±0.270 | 0.014±0.001 | 0.031±0.001^a^ |
|  |  | 0.2932 | 0.36 | <.0001 | 0.201 | 0.171 | 0.099 | 0.96 | 0.0507 | 0.002 |
| ***ZEB1*** | CC(277) | -8.613±0.385 | -17.161±0.467 | 0.000±0.000^b^ | 0.009±0.001 | 0.215±0.068^b^ | 2.591±0.307 | 6.504±0.376 | 0.016±0.001 | 0.027±0.002 |
| g.34066997C/G | CG(698) | -9.176±0.243 | -17.561±0.294 | 0.001±0.000^a^ | 0.008±0.000 | 0.416±0.043^a^ | 2.535±0.193 | 6.680±0.237 | 0.015±0.000 | 0.027±0.001 |
|  | GG(482) | -9.657±0.292 | -17.550±0.354 | 0.001±0.000^ab^ | 0.008±0.001 | 0.444±0.051^a^ | 2.500±0.233 | 6.746±0.286 | 0.014±0.001 | 0.031±0.001 |
|  |  | 0.094 | 0.748 | 0.023 | 0.849 | 0.017 | 0.972 | 0.875 | 0.399 | 0.058 |
| ***ZEB1*** | CC(292) | -8.670±0.377 | -17.206±0.453 | 0.000±0.000^b^ | 0.009±0.001 | 0.211±0.065^b^ | 2.570±0.298 | 6.543±0.366 | 0.016±0.001 | 0.026±0.002 |
| g.34063562C/G | CG(693) | -9.204±0.245 | -17.612±0.295 | 0.001±0.000^a^ | 0.008±0.000 | 0.422±0.042^a^ | 2.487±0.194 | 6.703±0.238 | 0.015±0.000 | 0.027±0.001 |
|  | GG(467) | -9.633±0.298 | -17.551±0.359 | 0.001±0.000^ab^ | 0.008±0.001 | 0.460±0.052^a^ | 2.535±0.236 | 6.741±0.290 | 0.014±0.001 | 0.031±0.001 |
|  |  | 0.132 | 0.748 | 0.025 | 0.805 | 0.007 | 0.97 | 0.908 | 0.501 | 0.089 |
| ***ZEB1*** | AA(481) | -9.561±0.295 | -17.551±0.353 | 0.001±0.000 | 0.008±0.001 | 0.359±0.051 | 2.443±0.232 | 6.446±0.286 | 0.015±0.001 | 0.028±0.001 |
| g.34110507T/A | TA(531) | -8.672±0.281 | -17.428±0.337 | 0.001±0.000 | 0.009±0.001 | 0.418±0.048 | 2.615±0.221 | 7.015±0.273 | 0.014±0.001 | 0.027±0.001 |
|  | TT(142) | -9.765±0.543 | -17.012±0.650 | 0.000±0.000 | 0.010±0.001 | 0.321±0.093 | 2.423±0.426 | 6.940±0.527 | 0.015±0.001 | 0.028±0.003 |
|  |  | 0.048 | 0.767 | 0.583 | 0.353 | 0.555 | 0.84 | 0.335 | 0.695 | 0.883 |
| ***ZEB1*** | AA(40) | -7.583±1.018 | -15.373±1.222 | 0.000±0.001^ab^ | 0.012±0.002^a^ | 0.068±0.178^ab^ | 3.086±0.810 | 6.700±0.994 | 0.015±0.002^ab^ | 0.027±0.005 |
| g.34061171T/C | GA(302) | -9.207±0.370 | -18.193±0.445 | 0.000±0.000^b^ | 0.007±0.001^b^ | 0.224±0.065^b^ | 2.998±0.295 | 5.988±0.362 | 0.016±0.001^a^ | 0.026±0.002 |
|  | GG(1071) | -9.313±0.197 | -17.369±0.236 | 0.001±0.000^a^ | 0.009±0.000^ab^ | 0.443±0.034^a^ | 2.399±0.157 | 6.896±0.192 | 0.014±0.000^b^ | 0.029±0.001 |
|  |  | 0.247 | 0.057 | 0.022 | 0.013 | 0.002 | 0.159 | 0.086 | 0.029 | 0.188 |

^1^AFC, age at the first service; AFS, age at the first calving; CE_C, calving ease in cows; CE_H, calving ease in heifers; ICF, the interval from calving to the first insemination; IFL_C, the interval from the first to last insemination in cows; IFL_H, the interval from the first to last insemination in heifers; SB_C, stillbirth in cows; SB_H, stillbirth in heifers.
