# Supplementary material for: A Post-GWAS Functional Analysis Confirming Effects of Three BTA13 Genes CACNB2, SLC39A12, and ZEB1 on Dairy Cattle Reproduction
Source: Front Genet. 2022 Jun 8;13:882951. doi: 10.3389/fgene.2022.882951 (PMC9216173; doi:10.3389/fgene.2022.882951)
Supplement: Supplementary file 4 [file Table3.DOCX]

**Table S3.** Primer pairs of *CACNB2*, *SLC39A12*, *ZEB1* and *GAPDH* genes are given for qRT-PCR.

| Gene | Primer Sequence（5’-3’） | | Length (bp) |
| --- | --- | --- | --- |
|  | Forward | Reverse |  |
| *GAPDH* | GGTGCTGAGTATGTGGTGGA | GGCATTGCTGACAATCTTGA | 180 |
| *CACNB2* | CTTCTATGCGACCTGTGG | AGCGGTGACCCTTGTG | 134 |
| *SLC39A12* | AGCACTGTGGCGGTGAC | GGTGGAGCAGAGCATC | 151 |
| *ZEB1* | ATATTGCTATACCTACCGTCAC | TTGCCTTTCATCCTGATTTCC | 232 |
