# Supplementary material for: A Post-GWAS Functional Analysis Confirming Effects of Three BTA13 Genes CACNB2, SLC39A12, and ZEB1 on Dairy Cattle Reproduction
Source: Front Genet. 2022 Jun 8;13:882951. doi: 10.3389/fgene.2022.882951 (PMC9216173; doi:10.3389/fgene.2022.882951)

**Figure S1:** Distributions of SNP clustering for the identified 18 SNPs in *CACNB2*, *SLC39A12* and *ZEB1*. It is obtained using KASP genotyping method. The red and the blue represents homozygous genotype; the green represents heterozygous genotype; the pink represents no or weak signal; the purple represents signal for no genotyping; and the black represents blank control.


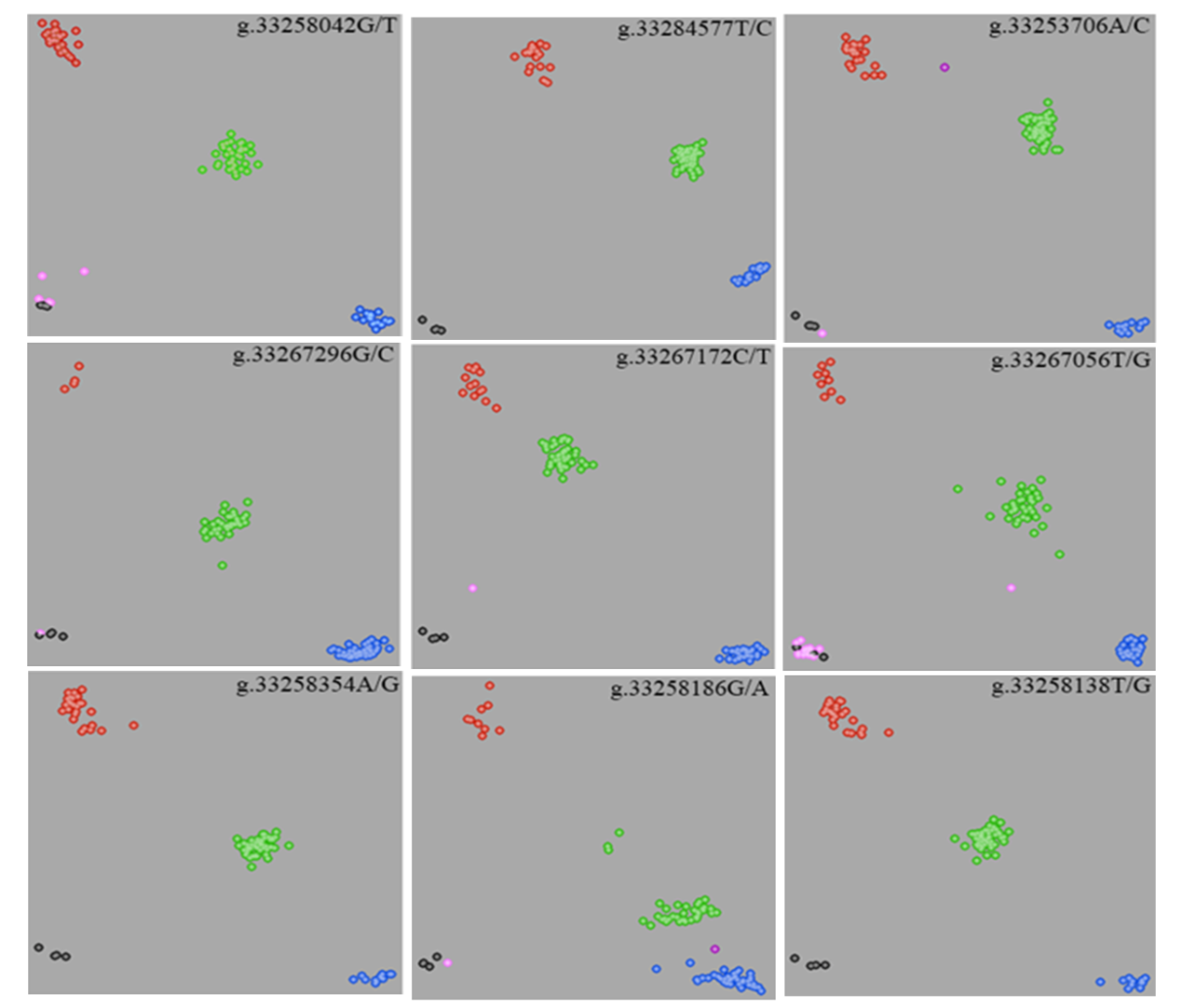


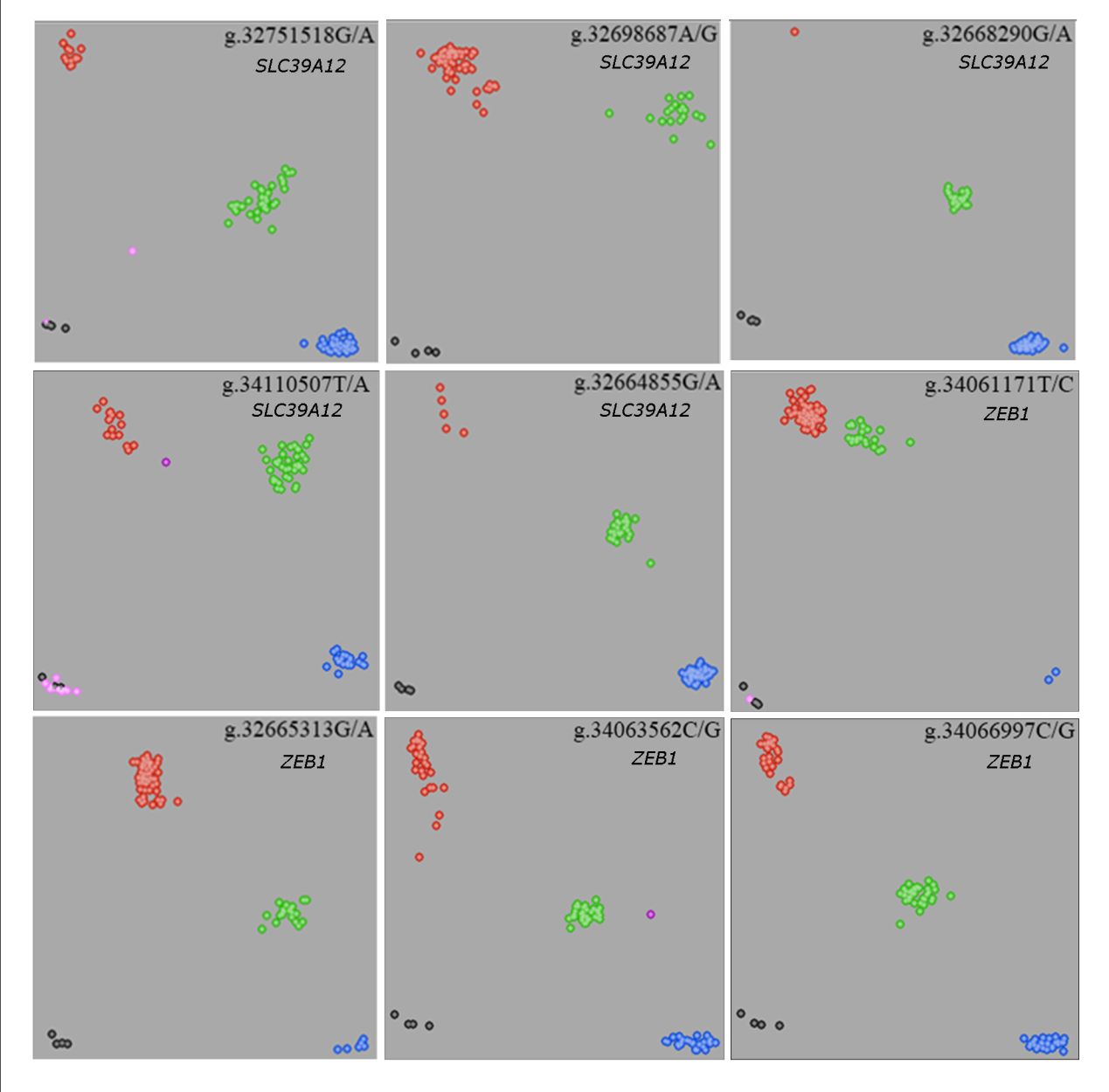

Supplement: Supplementary file 5 [file Table4.DOCX]
