# Supplementary material for: A Post-GWAS Functional Analysis Confirming Effects of Three BTA13 Genes CACNB2, SLC39A12, and ZEB1 on Dairy Cattle Reproduction
Source: Front Genet. 2022 Jun 8;13:882951. doi: 10.3389/fgene.2022.882951 (PMC9216173; doi:10.3389/fgene.2022.882951)
Supplement: Supplementary file 6 [file Table6.DOCX]

**Table S5.** The proportion of phenotypic variance explained by 18 SNPs in gene *CACNB2*, *SLC39A12* and *ZEB1* in Holstein cows.

| Gene | SNP | AFC | AFS | CE_C | CE_H | ICF | IFL_C | IFL_H | SB_C | SB_H |
| --- | --- | --- | --- | --- | --- | --- | --- | --- | --- | --- |
| *CACNB2* | g.33258042G/T | 0.005 | 0.002 | 0.001 | 0.000 | 0.001 | 0.002 | 0.001 | 0.001 | 0.003 |
| *CACNB2* | g.33258138T/G | 0.006 | 0.003 | 0.001 | 0.000 | 0.001 | 0.002 | 0.002 | 0.002 | 0.001 |
| *CACNB2* | g.33258186G/A | 0.006 | 0.002 | 0.001 | 0.000 | 0.000 | 0.001 | 0.010 | 0.000 | 0.007 |
| *CACNB2* | g.33253706A/C | 0.005 | 0.002 | 0.001 | 0.000 | 0.001 | 0.002 | 0.001 | 0.000 | 0.003 |
| *CACNB2* | g.33284577T/C | 0.005 | 0.000 | 0.002 | 0.000 | 0.001 | 0.002 | 0.002 | 0.002 | 0.000 |
| *CACNB2* | g.33258354A/G | 0.006 | 0.004 | 0.001 | 0.000 | 0.001 | 0.002 | 0.001 | 0.002 | 0.001 |
| *CACNB2* | g.33267056T/G | 0.011 | 0.004 | 0.002 | 0.000 | 0.001 | 0.004 | 0.007 | 0.001 | 0.000 |
| *CACNB2* | g.33267172C/T | 0.006 | 0.003 | 0.001 | 0.000 | 0.000 | 0.001 | 0.007 | 0.000 | 0.006 |
| *CACNB2* | g.33267296G/C | 0.002 | 0.000 | 0.000 | 0.000 | 0.001 | 0.001 | 0.000 | 0.001 | 0.008 |
| *SLC39A12* | g.32664855G/A | 0.001 | 0.001 | 0.000 | 0.000 | 0.001 | 0.000 | 0.001 | 0.000 | 0.002 |
| *SLC39A12* | g.32665313G/A | 0.001 | 0.000 | 0.000 | 0.000 | 0.001 | 0.000 | 0.001 | 0.000 | 0.003 |
| *SLC39A12* | g.32698687A/G | 0.000 | 0.002 | 0.000 | 0.001 | 0.000 | 0.001 | 0.007 | 0.000 | 0.001 |
| *SLC39A12* | g.32751518G/A | 0.001 | 0.000 | 0.001 | 0.000 | 0.001 | 0.001 | 0.000 | 0.000 | 0.038 |
| *SLC39A12* | g.32668290G/A | 0.000 | 0.005 | 0.000 | 0.000 | 0.000 | 0.000 | 0.000 | 0.000 | 0.001 |
| *ZEB1* | g.34110507T/A | 0.000 | 0.001 | 0.000 | 0.000 | 0.000 | 0.000 | 0.002 | 0.000 | 0.000 |
| *ZEB1* | g.34066997C/G | 0.003 | 0.001 | 0.000 | 0.000 | 0.001 | 0.000 | 0.000 | 0.001 | 0.012 |
| *ZEB1* | g.34063562C/G | 0.002 | 0.000 | 0.000 | 0.000 | 0.001 | 0.000 | 0.000 | 0.001 | 0.012 |
| *ZEB1* | g.34061171T/C | 0.001 | 0.001 | 0.000 | 0.000 | 0.002 | 0.001 | 0.003 | 0.004 | 0.009 |
| In total | | 0.061 | 0.031 | 0.011 | 0.001 | 0.014 | 0.020 | 0.045 | 0.015 | 0.107 |

^1^AFS, age at the first service; AFC, age at the first calving; CE_C, calving ease in cows; CE_H, calving ease in heifers; ICF, the interval from calving to the first insemination; IFL_C, the interval from the first to last insemination in cows; IFL_H, the interval from the first to last insemination in heifers; SB_C, stillbirth in cows; SB_H, stillbirth in heifers.
